# Diaphragm ultrasound as indicator of respiratory effort in critically ill patients undergoing assisted mechanical ventilation: a pilot clinical study

**DOI:** 10.1186/s13054-015-0894-9

**Published:** 2015-04-13

**Authors:** Michele Umbrello, Paolo Formenti, Daniela Longhi, Andrea Galimberti, Ilaria Piva, Angelo Pezzi, Giovanni Mistraletti, John J Marini, Gaetano Iapichino

**Affiliations:** Unità Operativa di Anestesia e Rianimazione, Azienda Ospedaliera San Paolo - Polo Universitario, Via A. Di Rudinì, 8-20142 Milano, Italy; Dipartimento di Fisiopatologia Medico-Chirurgica e dei Trapianti, Università degli Studi di Milano, Milano, Italy; Department of Pulmonary and Critical Care, University of Minnesota, Regions Hospital, St Paul, MN USA

## Abstract

**Introduction:**

Pressure-support ventilation, is widely used in critically ill patients; however, the relative contribution of patient’s effort during assisted breathing is difficult to measure in clinical conditions. Aim of the present study was to evaluate the performance of ultrasonographic indices of diaphragm contractile activity (respiratory excursion and thickening) in comparison to traditional indices of inspiratory muscle effort during assisted mechanical ventilation.

**Method:**

Consecutive patients admitted to the ICU after major elective surgery who met criteria for a spontaneous breathing trial with pressure support ventilation were enrolled. Patients with airflow obstruction or after thoracic/gastric/esophageal surgery were excluded. Variable levels of inspiratory muscle effort were achieved by delivery of different levels of ventilatory assistance by random application of pressure support (0, 5 and 15 cmH_2_O). The right hemidiaphragm was evaluated by B- and M-mode ultrasonography to record respiratory excursion and thickening. Airway, gastric and oesophageal pressures, and airflow were recorded to calculate indices of respiratory effort (diaphragm and esophageal pressure–time product).

**Results:**

25 patients were enrolled. With increasing levels of pressure support, parallel reductions were found between diaphragm thickening and both diaphragm and esophageal pressure–time product (respectively, R = 0.701, p < 0.001 and R = 0.801, p < 0.001) during tidal breathing. No correlation was found between either diaphragm or esophageal pressure–time product and diaphragm excursion (respectively, R = −0.081, p = 0.506 and R = 0.003, p = 0.981), nor was diaphragm excursion correlated to diaphragm thickening (R = 0.093, p = 0.450) during tidal breathing.

**Conclusions:**

In patients undergoing in assisted mechanical ventilation, diaphragm thickening is a reliable indicator of respiratory effort, whereas diaphragm excursion should not be used to quantitatively assess diaphragm contractile activity.

**Electronic supplementary material:**

The online version of this article (doi:10.1186/s13054-015-0894-9) contains supplementary material, which is available to authorized users.

## Introduction

The diaphragm is the main muscle that powers breathing. Impaired function of the diaphragm can lead to respiratory complications and often prolongs the duration of mechanical ventilation [1]. Conversely, mechanical ventilation itself may lead to diaphragm atrophy and dysfunction, which are well-recognized features of critically-ill patients [2,3]. Assisted mechanical ventilation, such as pressure-support ventilation (PSV), is widely used in critically ill patients with the aim of unloading the respiratory muscles while avoiding muscle atrophy [4]. In such modes, a variable amount of work is generated by the patient’s inspiratory muscles while the remainder is provided by the ventilator [5]. Low levels of assistance may lead to fatigue and discomfort, while over-assistance can generate patient-ventilator asynchrony [6] and mechanical ventilator-induced diaphragm dysfunction [7].

The relative contribution of the patient’s effort during assisted breathing is difficult to measure in clinical conditions, and the diaphragm, the major muscle of inspiratory function, is inaccessible to direct clinical assessment. Several methods have been used in the research setting to assess diaphragmatic contractile activity [8]. Among these, the standard reference is represented by the measurement of pleural (or esophageal (P_es_)) and abdominal (or gastric (P_ga_)) pressures and variables derived from those measurements [9]. However, such methods are still far from routine clinical practice, thus highlighting the need for simple and accurate methods to assess diaphragmatic performance in critically ill patients.

Bedside ultrasonography, which is already crucial in several aspects of critically illness [10], has been recently proposed as a simple, non-invasive method of quantification of diaphragmatic contractile activity [11]. Ultrasound can be used to determine diaphragm excursion [12,13], which may help to identify patients with diaphragm dysfunction [14]. Ultrasonographic examination can also allow for the direct visualization of the diaphragm thickness in its zone of apposition [15]. Thickening during active breathing has been proposed to reflect the magnitude of diaphragmatic effort, similarly to an ejection fraction of the heart [16].

The vast majority of reports addressing these ultrasonic indices were performed in spontaneously breathing patients [13,17-19], and the behavior of these measurements in patients undergoing mechanical ventilation has not yet been fully evaluated. The aim of the present study was to evaluate the performance of two ultrasonographic indices of diaphragm contractile activity (respiratory excursion and thickening) compared to gold-standard mechanical indices of inspiratory muscle effort.

## Materials and methods

### Ethics

This study was conducted in accordance with the amended Declaration of Helsinki. The local ethics committee (*Comitato Etico dell’Azienda Ospedaliera San Paolo di Milano*) approved the study protocol (#13864/2013) and patients gave their written consent to participate during pre-anesthetic assessment before surgery.

### Patients

Consecutive patients who were admitted to the ICU of a university hospital after major elective surgery were prospectively screened for enrolment between October 2013 and March 2014. Each patient had an orotracheal tube placed before surgery, and was mechanically ventilated in PSV mode (Evita XL, Drägerwerk AG, Lübeck, Germany) according to the clinical needs. Local guidelines for sedation of postoperative patients prescribe the use of an intravenous (iv) continuous infusion of propofol, starting at 1.5 mg/kg*h and titrated to obtain a Richmond agitation-sedation scale (RASS) score of 0/-1. Analgesia is provided as a 6- to 8-ml/h continuous epidural infusion of bupivacaine 0.125% + fentanyl 2-mcg/ml solution, aiming at a verbal numerical rating <4 or a behavioral pain scale <7. If epidural analgesia is not feasible, patients receive 0.5 to 1 mg/kg*h continuous iv infusion of morphine + iv acetaminophen 1 g three/four times per day.

Patients were enrolled when judged by the attending physician to be eligible for a test of weaning from mechanical ventilation, following the local weaning guidelines, that is, adequate cough, absence of excessive tracheobronchial secretion, clinical stability, heart rate (HR) <140/min, systolic blood pressure between 90 and 140 mmHg, arterial partial pressure of oxygen/inspired oxygen fraction (PaO_2_/FIO_2_) ≥150 mmHg, respiratory rate <35/min, maximal inspiratory pressure < −20 cmH2O, respiratory rate/tidal volume ratio <105 breaths/(min*l) [20].

Exclusion criteria were any of the following: hemodynamic instability requiring vasopressors, gas exchange impairment requiring positive end-expiratory pressure (PEEP) >10 cmH_2_O and/or FIO2 > 60% to obtain a PaO_2_ > 80 mmHg, pressure support (PS) level >20 cmH_2_O, body temperature >38°C or <35°C, deep sedation state (as defined by a RASS score < −1), intrinsic PEEP, or history of chronic obstructive pulmonary disease (COPD). Patients after thoracic, gastric or esophageal surgery were also excluded.

### Flow and pressure measurements

Flow was measured using a heated Fleisch number one pneumotachograph (Metabo SA, Epalinges, Switzerland) connected to a pressure transducer (T100209A, Edwards Lifesciences, Irvine, CA, USA), placed between the endotracheal tube and the ventilator Y connector. Airway pressure (Paw) was measured using a similar pressure transducer.

P_es_ and P_ga_ were measured using a double-balloon, graduated feeding catheter (NutriVent®, Mirandola, Modena, Italy) [21], which was positioned under general anesthesia before surgery for postoperative feeding. Both balloons were inflated with 2 ml of air and connected to an air-filled pressure transducer. To check the correct position of the esophageal balloon, a dynamic occlusion test was performed to assure that P_es_ was changing in concert with Paw when making efforts against a closed airway [22]. Transdiaphragmatic pressure (P_di_), the main determinant of the force generated by that muscle independently of any accessory muscle and elastic recoil of the system, was obtained by electronic subtraction of P_es_ from the P_ga_ signal [23] over ten consecutive breaths. Flow, Paw, P_es_ and P_ga_ were displayed on a dedicated multiparametric monitor (Datex Ohmeda S/5 Compact™, GE Healthcare, Little Chalfont, UK), collected at a sampling rate of 100 Hz, and recorded on a personal computer for subsequent analysis using dedicated software (Colligo, Elekton, Milan, Italy).

The esophageal and transdiaphragmatic pressure-time product (PTPes and PTPdi, respectively) per breath and per minute were obtained by measuring the area under the P_es_ or P_di_ signal from the onset of their negative (for P_es_) or positive (for P_di_) deflection to the end of inspiratory flow [24].

The airway pressure decrease in the first 100 ms after the onset of inspiration following an end-expiratory occlusion (P0.1) [25] was measured, reflecting the patient’s respiratory drive. The estimated pressure developed by the inspiratory muscles at the end of an inspiratory effort (Pmusc) [26], expressed as the Pmusc index (PMI) was also calculated as follows:

$$ \mathrm{P}\mathrm{M}\mathrm{I}=\mathrm{P}\mathrm{e}\mathrm{l},\mathrm{r}\mathrm{s}\mathrm{i}-\left(\mathrm{PEEP}+\mathrm{P}\mathrm{S}\right) $$

where Pel,rsi is the elastic recoil pressure of the respiratory system at the end of inspiration, measured as the airway pressure plateau value during an end-inspiratory occlusion maneuver, and PS is pressure support.

### Ultrasonographic measurements

Ultrasonography was performed by the same trained operator (DL) using an LogiQ7 (GE Healthcare, Little Chalfont, UK) equipped with a high resolution 10-MHz linear probe and a 7.5-MHz convex phased-array probe. Images were recorded for subsequent computer-assisted quantitative analysis performed by a trained investigator (AG), unaware of the ventilatory condition.

The convex probe was placed below the right costal margin along the mid-clavicular line, so that the ultrasound beam was perpendicular to the posterior third of the corresponding hemi-diaphragm, as previously described [13]. Patients were scanned along the long axis of the intercostal spaces, with the liver serving as an acoustic window. M-mode was then used to display diaphragm excursion, and three subsequent measurements were averaged. The values of diaphragm excursion in healthy individuals were reported to be 1.8 ± 0.3 cm during quiet breathing [13].

Diaphragm thickness was assessed in the zone of apposition of the diaphragm to the rib cage. The linear probe was placed above the right 10th rib in the mid-axillary line, as previously described [27]. The inferior border of the costophrenic sinus was identified as the zone of transition from the artifactual representation of normal lung to the visualization of the diaphragm and liver. In this area, the diaphragm is observed as a three-layered structure: a non-echogenic central layer bordered by two echogenic layers - the peritoneum and the diaphragmatic pleurae [27]. Three subsequent measures were averaged. The thickening fraction (TF) was calculated as follows:

$$ \mathrm{T}\mathrm{F}=\left(\mathrm{End}-\mathrm{inspiratory}\ \mathrm{thickness}\hbox{--} \mathrm{End}-\mathrm{expiratory}\ \mathrm{thickness}\right)/\mathrm{End}-\mathrm{expiratory}\ \mathrm{thickness}*100. $$

### Assessment of ultrasonographic indices reproducibility

Twenty recordings (from separate patients) were randomly selected to assess reproducibility: the same sets of recordings were analyzed twice by the same ultrasonographer (DL) and twice by a different ultrasonographer (AG). Repeated measurements obtained in each patient from the same ultrasonographer were used for intra-observer reproducibility, and measurements obtained in the same patient by the two ultrasonographers were used for inter-observer reproducibility.

### Study protocol

Patients were in the semi-recumbent position throughout the study. PEEP and FIO_2_ were set before the beginning of the study according to local guidelines, and were not modified throughout the study. Sedation was not modified either. In each patient, three levels of PS were applied in random sequence: 0, 5 and 15 cmH_2_O. Each level was applied for a minimum of 30 minutes, with the recording phase starting from the 20th minute to allow for a steady-state. Tidal Volume (Vt) and respiratory rate (RR) were recorded in each step, as well as P0.1 and PMI. During each of the three steps arterial blood gas analysis was performed, and arterial blood pressure (ABP) and HR were recorded. Diaphragm ultrasound scanning was also performed, and airway, esophageal and gastric pressure and flow were recorded. At the end of the recording phase PS was changed to that of the following step.

**Figure 1:**
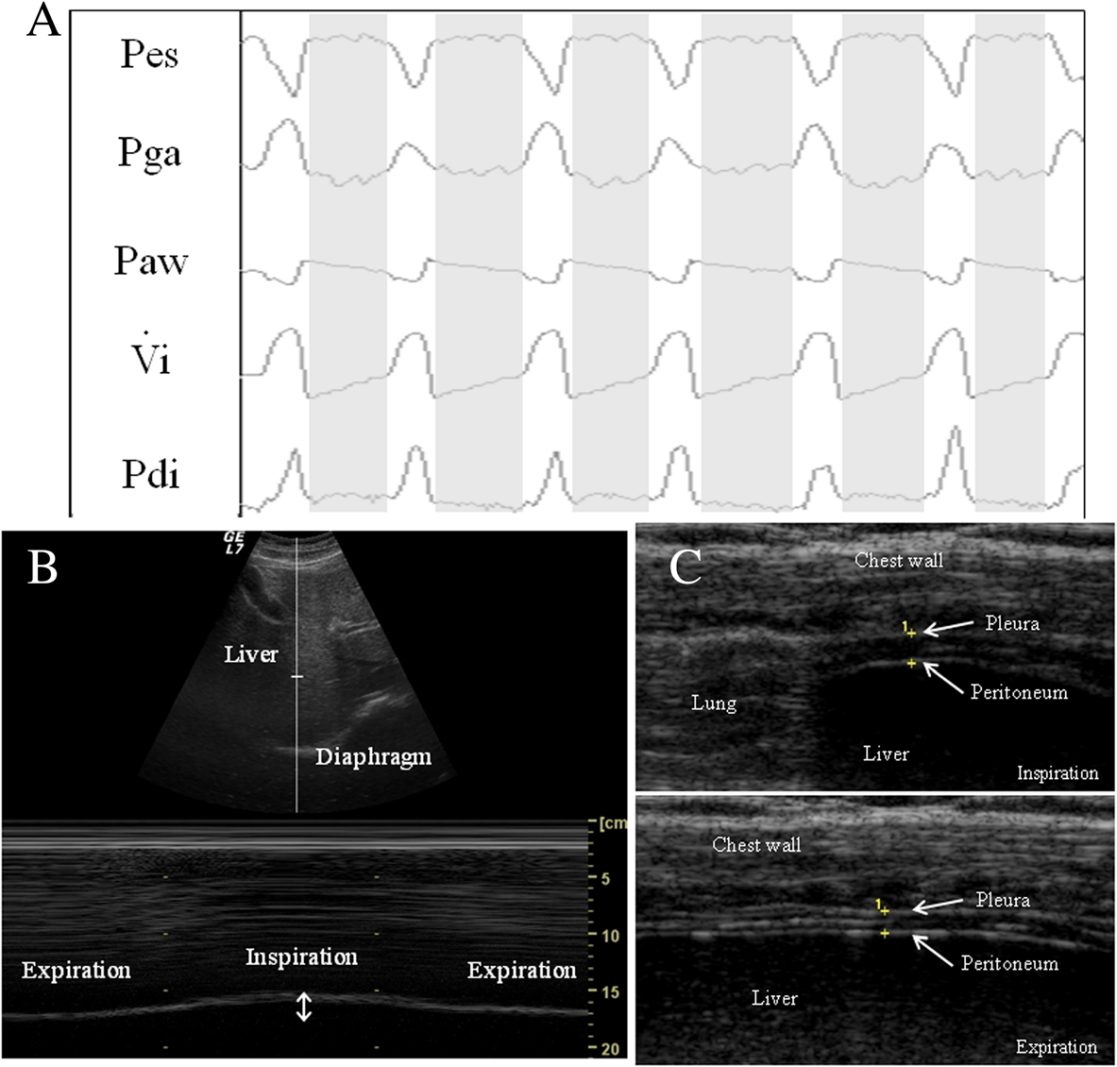
**Example of respiratory tracing and ultrasonographic measurements during the 0 cmH**_**2**_ **O pressure support step (PS0)** **.** **(A)** Sample recording of respiratory tracings during PS0: P_es_, esophageal pressure; P_ga_, gastric pressure; Paw, airway pressure; Vi, respiratory flow; P_di_, transdiaphragmatic pressure. White area represents inspiration and gray area depicts expiration. **(B)** Ultrasonographic view of diaphragmatic excursion during breathing in B-mode (upper) and M-mode (lower). **(C)** Ultrasonographic view of diaphragm in the zone of apposition during inspiration (upper) and expiration (lower); the diaphragm is identified as a three-layer structure (non-echogenic central layer bordered by two echogenic layers, the peritoneum and the diaphragmatic pleurae) as indicated by yellow crosses.

The protocol was allowed to be stopped, and PS increased to the pre-study value to allow patients to rest for at least 30 minutes whenever they developed one of the following signs of respiratory distress: RR >35 breaths/min, a peripheral pulse oximeter oxygen saturation (SpO2) < 90%, HR >140 beats/min or variation >30% from baseline, ABP >180 mmHg, diaphoresis or anxiety. Conversely, PS level was lowered to the value before the beginning of the study if PS 15 cmH_2_O was not tolerated for over-assistance (cough, Vt >15 mL/kg, and/or absence of inspiratory efforts >15 s). Figure 1 shows an example of respiratory tracing and ultrasonographic measurements during the 0 cmH_2_O pressure support step (PS0).

### Statistical analysis

Data were analyzed using Stata 11 (StataCorp LP, College Station, TX, USA). Normality was assessed by the Shapiro-Francia test. Results are reported as mean ± standard deviation if normally distributed, or median (25 to 75th percentiles) otherwise. Comparison of variables over the different steps was performed by repeated-measures analysis of variance or the Friedman test with post-hoc comparison, as needed. Correlation was assessed using Pearson’s or Spearman’s method according to the distribution of the variable. To deal with the longitudinal structure of our dataset (patients with repeated measurements over time), regression analysis was conducted by building a linear mixed model for repeated measures based on each patient. In this case, the extent of the association between variables was expressed as the *b* coefficient. Reproducibility was expressed by the intra-class correlation coefficient [28] and the coefficient of repeatability [29]. The latter was calculated as twice the standard deviation of the differences in repeated measurements. Two-tailed *P*-values <0.05 were considered statistically significant.

## Results

### Patients’ characteristics

During the study period, 114 patients met the inclusion criteria; 79 were excluded from the study (COPD 36, thoracic surgery 18, gastric surgery 14, oesophageal surgery 11). Of the 35 remaining patients 10 more were excluded because of technical/organizational issues (unavailability of the ultrasound machine, lack of research personnel). Twenty-five consecutive patients were then enrolled; all presented an adequate ultrasonographic window. Patients’ demographics and clinical data are shown in Table 1. PEEP and FIO_2_ were set by the attending physician and remained constant during the whole study (as per study protocol). The values of PEEP and FIO_2_ were 6.8 ± 1.6 cmH_2_O and 0.53 ± 0.11, respectively. All patients tolerated the study protocol well, and none developed signs of respiratory distress or over-assistance. All patients were successfully weaned from mechanical ventilation and discharged alive.

**Table 1:** **Patients' characteristics at baseline**

**Characteristic**	**Value**
**Age, years**	71 (51; 78)
**Height, cm**	169 ± 7
**Weight, kg**	74 (72; 85)
**Body mass index, kg/cm** ^**2**^	26.4 (23.9; 30.1)
**Sex**	
Male	21 (84%)
Female	4 (16%)
**Simplified acute physiology score 2**	29.2 ± 7.6
**Type of surgery**	
Abdominal	10 (40%)
Vascular	5 (20%)
Endocrine	4 (16%)
Urologic	3 (12%)
Others	3 (12%)
**Richmond agitation-sedation scale score**	
−1	9 (36%)
0	16 (64%)
**Length of stay, days**	2.6 ± 2.2
**Length of mechanical ventilation, days**	1.4 ± 0.9

Results are presented as median (IQR), mean ± SD, or number (percent).

### Reproducibility of ultrasound and esophageal pressure measurements

The calibration procedure for the esophageal balloon was strongly correlated between Paw and P_es_ during the dynamic occlusion test (slope = 0.934, *P* <0.001). Adequate intra- and interobserver reproducibility for diaphragmatic thickness and respiratory displacement was found, as shown in Additional file 1.

### Effect of variable levels of pressure support on ventilation and hemodynamics

Table 2 reports respiratory and hemodynamic parameters during the three steps of the study. As expected, Vt increased with increasing levels of support, whereas RR decreased. Neither global hemodynamics, nor gas exchange were modified with increasing levels of ventilator assistance.

**Table 2:** **Respiratory and hemodynamic data during the three steps of the study**

	**PS15**	**PS5**	**PS0**	***P***
**Tidal volume, ml**	836 ± 250	496 ± 210	456 ± 182	<0.001
**Respiratory rate, min** ^**−1**^	10.7 ± 4.3	16.0 ± 6.8	16.7 ± 7.6	0.003
**Minute ventilation, l/min**	8.2 ± 2.1	7.4 ± 2.5	6.8 ± 2.1	0.119
**Mean airway pressure, cmH**_**2**_ **O**	11.8 ± 2.9	8.7 ± 2.0	7.6 ± 1.9	<0.001
**Mean arterial pressure, mmHg**	77.9 ± 8.8	78.8 ± 10.6	80.2 ± 11.4	0.751
**Heart rate, min** ^**−1**^	63.9 ± 19.1	65.7 ± 19.4	66.5 ± 21.0	0.899
**pH**	7.39 ± 0.05	7.37 ± 0.04	7.36 ± 0.04	0.090
**PaO**_**2**_ **, mmHg**	176.7 ± 48.3	179.3 ± 50.0	175.2 ± 61.9	0.967
**PaCO**_**2**_ **, mmHg**	42.2 ± 5.3	43.8 ± 4.4	45.1 ± 5.1	0.147
**Base excess, mmol/L**	1.0 ± 2.9	0.7 ± 2.9	0.6 ± 3.0	0.899

Results are expressed as mean ± SD. PS, pressure support; PaO2, partial pressure of oxygen in arterial blood; PaCO2, partial pressure of carbon dioxide in arterial blood.

**Figure 2:**
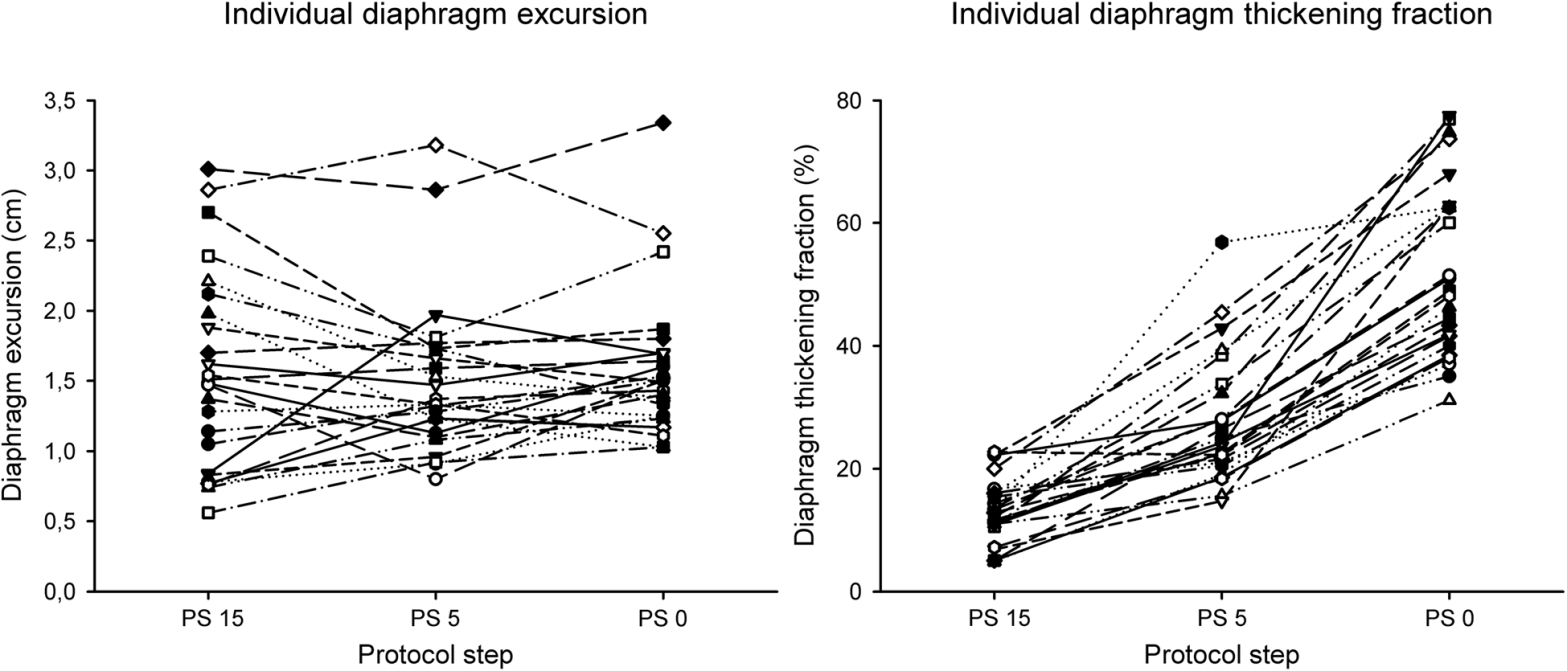
**Individual patient data for diaphragm excursion and thickening fraction in the different steps of the study.** PS, pressure support.

Figure 2 shows individual patient data for diaphragm excursion and thickening fraction in the different steps of the study.

### Ultrasonography and inspiratory effort measurements

Measurements of inspiratory effort and ultrasound measurements are reported in Table 3. Increasing levels of support were associated with significant decreases in P0.1 and PMI, as well as with significantly decreased esophageal pressure-time product (PTPes) and esophageal pressure-time product transdiaphragmatic pressure (PTPdi), either per breath or per minute.

**Table 3:** **Ultrasonographic and pressure measurements during the three steps of the study**

	**PS15**	**PS5**	**PS0**	***P***
**PMI, cmH**_**2**_ **O**	−3.65 ± 5.31	2.58 ± 4.86	6.61 ± 4.36	<0.001
**P0.1, cmH**_**2**_ **O**	0.30 (0.20; 0.70)	0.80 (0.50; 1.20)	1.80 (1.20; 2.80)	<0.001
**PTPes/breath, cmH**_**2**_ O* sec	0.50 (0.12; 0.82)	2.11 (0.96; 3.72)	4.28 (2.86; 6.70)	<0.001
**PTPdi/breath, cmH**_**2**_ O* sec	1.12 (0.17; 1.69)	2.73 (1.63; 3.80)	4.48 (3.30; 7.48)	<0.001
**PTPes/min, cmH**_**2**_ O* sec/min	4.78 (0.76; 8.98)	26.10 (16.20; 44.69)	53.31 (39.30; 77.11)	<0.001
**PTPdi/min, cmH**_**2**_ O* sec/min	10.60 (1.09; 23.03)	36.15 (30.06; 62.66)	57.12 (54.39; 109.84)	<0.001
**ΔPes, cmH**_**2**_ **O**	3.79 ± 2.65	−2.36 ± 3.03	−4.85 ± 3.67	<0.001
**ΔPdi, cmH**_**2**_ **O**	2.78 (1.09; 4.22)	−1.65 (−5.03; −0.25)	−3.54 (−7.57; −1.54)	<0.001
**Thickening fraction, %**	13.0 ± 5.2	28.2 ± 9.9	52.7 ± 15.9	<0.001
**Excursion, cm**	1.48 (0.84; 1.98)	1.33 (1.13; 1.73)	1.50 (1.25; 1.70)	0.797

Results are expressed as mean ± SD or median (IQR). PS, pressure support; PMI, estimated pressure developed by the inspiratory muscles at the end of an inspiratory effort (Pmusc) index; P0.1, airway occlusion pressure in the first 100 ms; PTPes, esophageal pressure-time product; PTPdi diaphragmatic pressure-time product; ΔPes, inspiratory variation of esophageal pressure; ΔPdi, inspiratory variation of transdiaphragmatic pressure.

**Figure 3:**
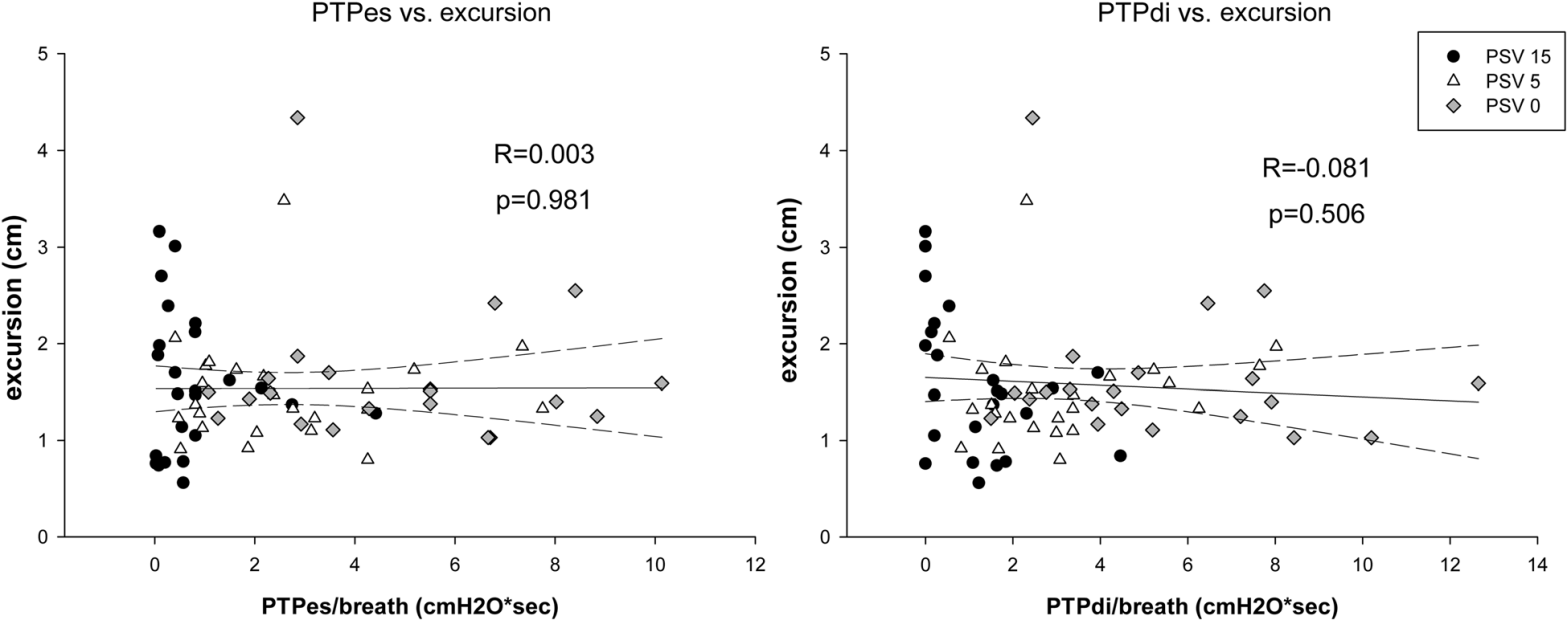
**Correlation between excursion and esophageal and diaphragmatic pressure-time product.** PTPes, esophageal pressure-time product per breath; PTPdi, diaphragmatic pressure-time product per breath; PSV, pressure support ventilation.

Ultrasonographic TF of the diaphragm also significantly changed and decreased with increasing ventilator support, whereas diaphragmatic excursion was unaltered. No correlation was found between diaphragm excursion and PTPes or PTPdi (*b* coefficient = 0.032, *P* = 0.900 and −0.005, p = 0.720, respectively) (Figure 3), nor was this index correlated with PMI (*b* = −0.002, *P* = 0.872), P0.1 (*b* = 0.002, *P* = 0.945), RR (*b* = −0.004, *P* = 0.754) or Vt (*b* = 0.001, *P* = 0.991).

**Figure 4:**
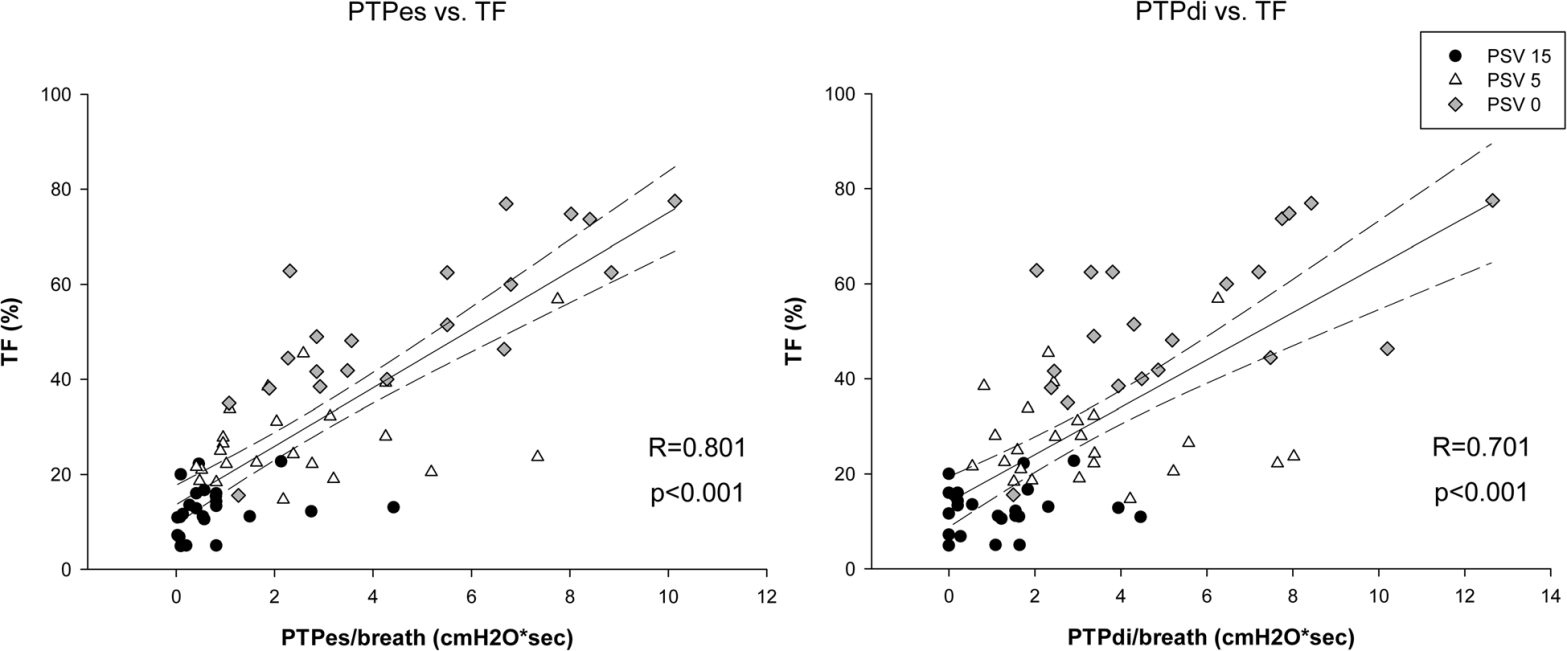
**Correlation between thickening fraction and esophageal and diaphragmatic pressure-time product.** PTPes, esophageal pressure-time product per breath; PTPdi, diaphragmatic pressure-time product per breath; TF, thickening fraction; PSV, pressure support ventilation.

Diaphragm TF significantly correlated with PTPes and PTPdi (*b* coefficient = 4.459, *P* <0.001 and 2.322, *P* <0.001, respectively) (Figure 4), as well as with PMI (*b* = 1.140, *P* = 0.001) and P0.1 (*b* = 5.522, *P* <0.001). Negative correlation was found between TF and Vt (*b* = −0.030, *P* <0.001). No correlation was found between diaphragm TF and excursion (*b* = 0.002, *P* = 0.488).

### Effect of PEEP on diaphragm thickness and thickening

Because in our patient population PEEP was selected by the attending physician based on clinical criteria and thus, could differ among patients, we analyzed the effect of different levels of PEEP on the values of diaphragm end-expiratory thickness and thickening fraction at every PS level. No significant correlation was found between TF and the level of PEEP (PS15: *R* = 0.031, *P* = 0.884; PS5: *R* = 0.168, *P* = 0.422; PS0: R = 0.254, *P* = 0.253). Similarly, no relationship was found between diaphragm thickness at end-expiration and the level of PEEP (PS15: *R* = 0.033, *P* = 0.875; PS5: *R* = −0.021, *P* = 0.922; PS0: *R* = −0.097, *P P* = 0.668).

## Discussion

The main findings of this work can be summarized as follows: in a population of post-surgical patients during assisted tidal breathing, a parallel reduction was found between ultrasonographic assessment of diaphragm thickening and indices of respiratory muscle effort when different amounts of respiratory effort were achieved by titration of pressure support. On the contrary, no correlation was found between indices of muscle effort and diaphragm excursion, nor was the latter correlated to diaphragm thickening.

PSV is a commonly used ventilation mode, both as stand-alone ventilatory support in acute respiratory failure and/or during the weaning phase of mechanical ventilation [30]. However, the rationale and the clinical guidelines for its use are still rather undefined. The aim of this mode is to unload the respiratory muscles, preserving spontaneous contraction and thus avoiding atrophy [31]. However, low levels of support may still lead to fatigue and discomfort, whereas over-assistance can generate patient-ventilator asynchrony [6] and mechanical ventilation-induced diaphragmatic dysfunction [7].

Various non-invasive indices have been suggested to adjust the level of support, such as clinical assessment of accessory muscle activity [32], RR and Vt [31], the assessment of respiratory drive (P0.1) [25], or the pressure developed by the inspiratory muscles (PMI) [26]. However, they either lack adequate sensitivity/specificity, or require a cooperative patient. Our aim was to evaluate the ability of ultrasonographic indices to assess diaphragm contractile activity.

As in other similar physiologic studies [26,33,34] we varied the level of PS to explore the behavior of the ultrasonographic indices over a range of conditions of respiratory muscle load. The upper level of PS was set at 15 cmH_2_O, as that value was shown to take over the major part of the work of breathing in postoperative patients without pre-existing pulmonary diseases [35]. We evaluated the relationship between ultrasonographic indices of diaphragm contractile activity with well-validated invasive and non-invasive indices of inspiratory effort. As expected, respiratory rate decreased and tidal volume increased with increasing levels of support [36], with minimal changes in minute ventilation and no effect on global hemodynamics [37].

Diaphragm excursion has been extensively studied as an index of diaphragmatic contractile activity [12,13,18,19]. However, those studies were all performed in spontaneously breathing patients, whereas the role of excursion in the functional evaluation of diaphragm contractile activity during assisted mechanical ventilation is far less clear. In the present study, this index proved uncorrelated to any of the other indices of inspiratory effort taken into account. In fact, diaphragm excursion during an assisted breath represents the sum of two forces acting in the same direction: the force of the diaphragm contraction by itself, and the passive displacement of the diaphragm by the pressure provided by the ventilator. With increasing levels of PS, the diaphragm is unloaded and an increasing part of the work of breathing is performed by the ventilator. This resulted in a similar degree of diaphragm excursion despite significantly different levels of muscle effort. In this case, there is no means to distinguish which part of displacement is passive, and which is active. As a consequence, during PSV, excursion might not represent a reliable index for the monitoring of diaphragm contractile activity and for the assessment of inspiratory effort.

As expected for our case-mix, we observed a normally functioning diaphragm in all patients, as testified by the absence of need for prolonged mechanical ventilation or difficult weaning and a thickening fraction well above the 20% cutoff during PS0 [38]. However, an excursion only slightly higher than 1 cm was our median value, whereas values <1 cm during quiet breathing are considered as indicating diaphragm dysfunction [12,13,39]. This may suggest that the suggested cutoffs for diaphragm dysfunction, as far as diaphragmatic excursion is concerned, should not be considered during assisted mechanical ventilation and should be reassessed in further studies.

One of our hypotheses was that diaphragm thickening would be a more accurate index of diaphragm contractile activity than excursion during pressure-support ventilation, as thickening should only be influenced by active contraction. We found a significant correlation between thickening fraction and both PTPes and PTPdi. Although most evidence regarding the role of ultrasonographic assessment of diaphragm thickening relates to studies performed on spontaneously breathing patients [13,17-19], it was recently demonstrated how this index might be useful for the assessment of the effort of breathing during non-invasive PSV [33].

Another interesting finding of our study is the negative correlation between TF and Vt. In spontaneously breathing patients, TF was shown to be positively related to tidal volume [15]. However, during pressure support breathing, Vt depends on the balance between the force provided by the patient and the ventilator. Moreover, in our study, Vt was significantly higher at high levels of pressure support, when respiratory effort was low. This negative relationship might then simply be representative of the reduced muscle work at high levels of support. Thickness measurements during spontaneous breathing may be influenced by lung volume in a non-linear relationship [27], and diaphragmatic thickening was shown to be more pronounced above 50% of vital capacity [40]. Again, these data come from studies performed on spontaneously breathing subjects. In our population, we were unable to find any difference between thickness or diaphragm thickening and the level of PEEP. However, this may depend on the relatively low level of PEEP applied in the patients we enrolled, while we have no data on absolute lung volume as these measurements were not performed.

On the other hand, an apparently counterintuitive finding was that despite a relevant increase in TV between PS0 and PS15, diaphragmatic excursion remained almost constant. The most likely explanation is that with higher levels of PS a higher portion of the TV is distributed to the non-dependent lung region due to better compliance of this area, as recently shown with electrical impedance tomography analysis [41,42]. Another possible explanation is that during PS15 patients’ respiratory muscles could have been over-assisted and their diaphragm might have only been triggering the ventilator and then relaxed, so that they were passively ventilated for the large majority of the inspiratory phase and the diaphragm passively displaced downward, and again TV was distributed to non-dependent areas characterized by higher regional compliance [43].

A common drawback of ultrasonography is its operator dependence. We therefore assessed the intraobserver and interobserver reproducibility of both diaphragmatic excursion and thickness. We found an overall good repeatability of our assessments, with intra-class correlation coefficients well above 0.75, usually considered to indicate good agreement [44]. The coefficient of repeatability, that is, the smallest significant difference between repeated measurements [29], was lower for the measurement of excursion than for that of thickening. This suggests, as others have pointed out [33], that the reproducibility of the latter may be difficult to achieve in some patients, possibly because a stable image is not simple to obtain, especially in the case of increased respiratory workload. Nevertheless, our results are not different from those reported in studies with spontaneously breathing patients [45], or patients undergoing non-invasive ventilation [33].

Some other limitations of this study need to be acknowledged. We studied a relatively small population; however, this was comparable with that included in similar physiological studies [26,34]. Furthermore, we only included patients after major elective surgery. Another limitation comes from the patients’ selection criteria: it is unknown if these results can be translated to patients with intrinsic PEEP or COPD because this was an exclusion criterion. We only assessed the right hemi-diaphragm because its visualization is easier as compared to the left side where imaging is often impeded by gastric and intestinal gas interposition. Again, this limitation is common to other studies on ultrasonographic assessment of diaphragmatic contractile activity [33]. A limitation of diaphragm sonography is a poor acoustic window; this is reported to occur in a minority of patients, varying between 2 and 10% [14,33]. In accordance with other studies, we were able to complete the evaluation in all of our patients. Finally, we only included patients with PEEP <10 cmH_2_O. To date, there is no evidence about feasibility and accuracy of diaphragmatic ultrasonography in the presence of elevated levels of PEEP, when the increase in lung volume might cause a displacement of the superior edge of the zone of apposition [46].

## Conclusions

In conclusion, we found that in postoperative patients undergoing assisted spontaneous breathing diaphragm thickening was a good indicator of changes of inspiratory muscle effort in response to modifications of the PS level. We suggest that the use of diaphragm excursion is of little help during PSV and should not be used to quantitatively assess diaphragm contractile activity. Further studies are warranted to assess if this holds true in a greater number of patients with different diseases.

## Key messages

- Diaphragm ultrasonography is a simple, non-invasive method of quantification of diaphragm contractile activity.
- During spontaneous ventilation, the assessment of diaphragm respiratory excursion may help to identify patients with diaphragm dysfunction; however, during assisted mechanical ventilation, the role of excursion in the functional evaluation of diaphragm contractile activity is far less clear. Little is known about the role of diaphragm thickening.
- In this physiologic, clinical pilot study we found that diaphragm excursion was not correlated to any index of muscle effort under varying levels of muscle loading; we also found that diaphragm thickening was a good indicator of changes of inspiratory muscle effort in response to modifications of the support level.
- Monitoring of diaphragm contractile activity during the weaning phase should be performed with diaphragm thickening, and the suggested cutoffs for diaphragm dysfunction, as far as diaphragmatic excursion is concerned, should not be considered valid during assisted mechanical ventilation.

## Additional file

Additional file 1:
**Intra- and interobserver reliability of ultrasonographic measurements of diaphragm function.** Intraobserver reliability is provided in **Table S1**, and interobserver reliability is shown in **Table S2**.

## Abbreviations

ABP: arterial blood pressure
COPD: chronic obstructive pulmonary disease
HR: heart rate
iv: intravenous
P0.1: airway pressure drop in the first 100 ms
PaO_2_/FiO_2_: arterial partial pressure of oxygen/inspired oxygen fraction
Paw: airway pressure
P_di_: transdiaphragmatic pressure
PEEP: positive end-expiratory pressure
P_es_: esophageal pressure
P_ga_: gastric pressure
PMI: estimated pressure developed by the inspiratory muscles at the end of an inspiratory effort (Pmusc) index
PS: pressure support
PSV: pressure-support ventilation
PTPdi: transdiaphragmatic pressure-time product
PTPes: esophageal pressure-time product
RASS: Richmond agitation-sedation scale
RR: respiratory rate
TF: thickening fraction
Vt: tidal volume
WOB: work of breathing
