# Supplementary material for: Diaphragm ultrasound as indicator of respiratory effort in critically ill patients undergoing assisted mechanical ventilation: a pilot clinical study
Source: Crit Care. 2015 Apr 13;19(1):161. doi: 10.1186/s13054-015-0894-9 (PMC4403842; doi:10.1186/s13054-015-0894-9)
Supplement: Additional file 1: — Intra- and interobserver reliability of ultrasonographic measurements of diaphragm function. Intraobserver reliability is provided in Table S1, and interobserver reliability is shown in Table S2. [file 13054_2015_894_MOESM1_ESM.pdf]

Diaphragm ultrasound as indicator of respiratory effort in critically ill patients undergoing assisted mechanical ventilation: a pilot clinical study

Michele Umbrello[a], Paolo Formenti[a], Daniela Longhi[b], Andrea Galimberti[b], Ilaria Piva[b], Angelo Pezzi[a], Giovanni Mistraletti[a,b], John J. Marini[c], Gaetano Iapichino[a,b].

From the: [a]Unità Operativa di Anestesia e Rianimazione, Azienda Ospedaliera San Paolo - Polo Universitario, Milano; [b]Dipartimento di Fisiopatologia Medico-Chirurgica e dei Trapianti, Università degli Studi di Milano; [c] Department of Pulmonary and Critical Care, University of Minnesota, Regions Hospital, St Paul, MN, USA

Corresponding author and address for reprints:

Michele Umbrello, MD

UO Anestesia e Rianimazione

A.O. San Paolo - Polo Universitario

Via A. Di Rudinì, 8 - 20142 Milano - Italy

Email michele.umbrello@ao-sanpaolo.it

Table S1 – Intraobserver reliability of ultrasonographic measurements of diaphragm function

End Inspiratory diaphragmatic thickness (cm)

Observer	Mean	Range	IntraCC	Repeatability coeff (cm)
1 (D.L.)	0.395	0.16; 0.59	0.877	0.041
2 (A.G.)	0.396	0.18; 0.57	0.876	0.039

End Expiratory diaphragmatic thickness (cm)

Observer	Mean	Range	IntraCC	Repeatability coeff (cm)
1 (D.L.)	0.282	0.15; 0.45	0.985	0.030
2 (A.G.)	0.280	0.17; 0.43	0.986	0.020

Thickening fraction (%)

Observer	Mean	Range	IntraCC	Repeatability coeff (%)
1 (D.L.)	42.4	3; 84	0.986	10.6
2 (A.G.)	44.5	2; 83	0.990	8.89

Respiratory diaphragmatic displacement (cm)

Observer	Mean	Range	IntraCC	Repeatability coeff (cm)
1 (D.L.)	1.97	0.80; 3.18	0.998	0.080
2 (A.G.)	1.91	0.74; 3.18	0.999	0.068

IntraCC: Intra-class correlation coefficient; Repeatability coeff: repeatability coefficient

Table S2 - Interobserver reliability of ultrasonographic measurements of diaphragm function

End Inspiratory diaphragmatic thickness (cm)

Mean	Range	InterCC	Repeatability coefficient (cm)	Difference between the two observers	
				Mean	95% CI
0.39	0.17; 0.58	0.965	0.065	-0.001	-0.081; 0.079

End Expiratory diaphragmatic thickness (cm)

Mean	Range	InterCC	Repeatability coefficient (cm)	Difference between the two observers	
				Mean	95% CI
0.28	0.16; 0.43	0.989	0.023	-0.006	-0.113; 0.001

Thickning fraction (%)

Mean	Range	InterCC	Repeatability coefficient (%)	Difference between the two observers	
				Mean	95% CI
41.5	4.6; 82.1	0.936	17.9	0.89	-4.58; 6.36

Respiratory diaphragmatic displacement (cm)

Mean	Range	InterCC	Repeatability coefficient (%)	Difference between the two observers	
				Mean	95% CI
1.94	0.77; 3.18	0.988	0.256	0.065	-0.48; 0.62

InterCC: Inter-class correlation coefficient;; 95% CI: 95% confidence interval
